# Intermittent fasting and immunomodulatory effects: A systematic review

**DOI:** 10.3389/fnut.2023.1048230

**Published:** 2023-02-28

**Authors:** Zhangyuting He, Haifeng Xu, Changcan Li, Huayu Yang, Yilei Mao

**Affiliations:** ^1^Peking Union Medical College, Chinese Academy of Medical Sciences and Peking Union Medical College, Beijing, China; ^2^Department of Liver Surgery, Peking Union Medical College (PUMC) Hospital, PUMC and Chinese Academy of Medical Sciences (CAMS), Beijing, China

**Keywords:** intermittent fasting, immune system, immunomodulatory effect, metabolic syndrome, obesity

## Abstract

**Introduction:**

strategy of periodic food restriction and fixed eating windows, could beneficially modify individuals by losing body weight, regulating glucose or lipid metabolism, reducing blood pressure, and modulating the immune system. Specific effects of IF and its mechanisms have not yet been assessed collectively. Thus, this systematic review aims to summarize and compare clinical trials that explored the immunomodulatory effects of IF.

**Methods:**

After screening, 28 studies were included in this systematic review.

**Results:**

In addition to weight loss, IF could benefit health subjects by strengthening their circadian rhythms, migrating immune cells, lower inflammatory factors, and enriching microbials. In addition of the anti-inflammatory effect by regulating macrophages, protection against oxidative stress with hormone secretion and oxidative-related gene expression plays a key beneficial role for the influence of IF on obese subjects.

**Discussion:**

Physiological stress by surgery and pathophysiological disorders by endocrine diseases may be partly eased with IF. Moreover, IF might be used to treat anxiety and cognitive disorders with its cellular, metabolic and circadian mechanisms. Finally, the specific effects of IF and the mechanisms pertaining to immune system in these conditions require additional studies.

## 1. Introduction

**Figure 1:**
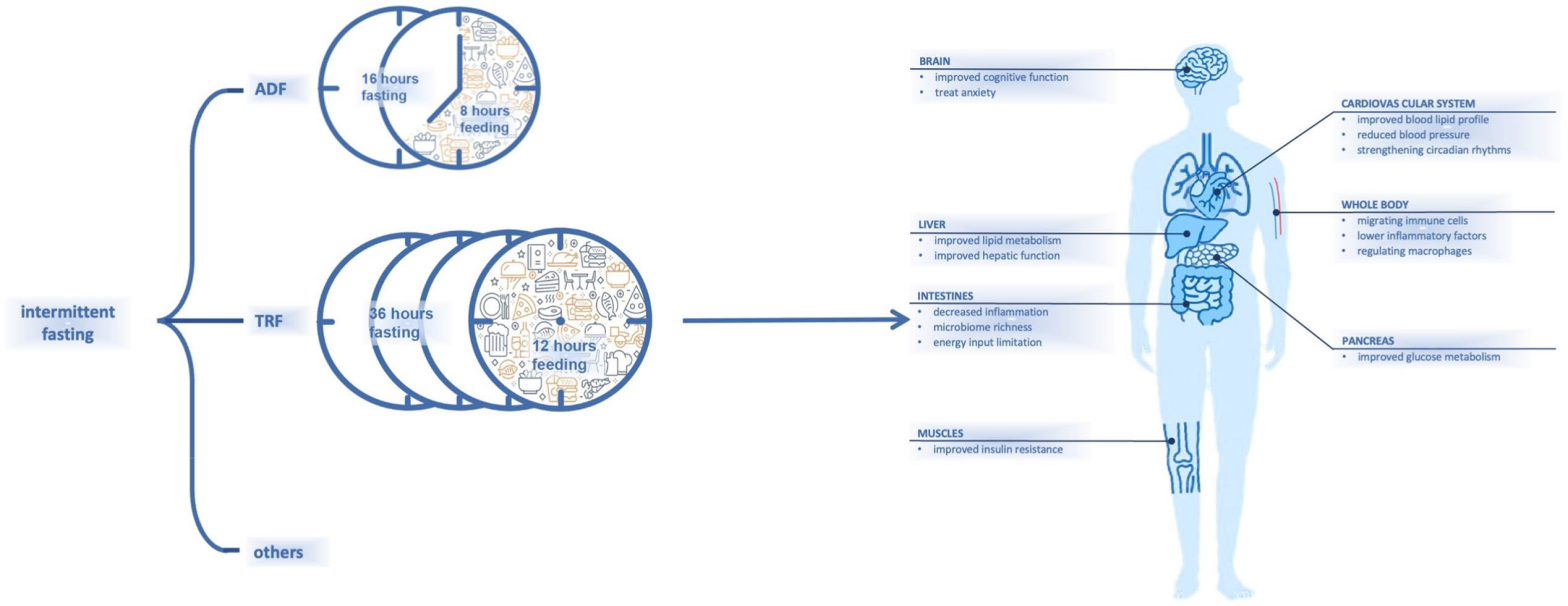
Content and presumed influences on body of intermittent fasting.

Fasting has recently received increasing attention for its advantages on body health (1). Dietary habits that involve fat-rich foods and snacks may lead to chronic diseases (2). Intermittent fasting (IF), as a dieting strategy, combines periodic energy restriction and fixed-duration eating windows (3). Different types of IF that incorporate varied combinations of fasting and eating windows have been proposed; examples include alternate-day fasting (36 h of fasting and 12 h of *ad libitum* eating) (4) and time-restricted fasting (16 h of fasting and 8 h of *ad libitum* eating) (5) (Figure 1).

It has been shown that IF is effective for decreasing body weight (6), and it can help to regulate glucose or lipid metabolism and reduce blood pressure (7) (Figure 1). In one study, numerous subjects with metabolic syndrome experienced improvements in lipid and glucose metabolism after IF (8). Another study had also noted that healthy and lean people may experience metabolic improvements by resetting their dietary intake with a schedule of fasting and eating (9). As studies of additional parameters including pre-inflammatory markers have been conducted, other effects of fasting have been observed.

One area of great interest is the influence of fasting on the immune system, which responds to stressful and harmful events in the body (10). The immune system can be regulated by weight reduction; changes in lipid and glucose metabolism; and other processes, such as circadian rhythm changes (10–14). Whether the influence of fasting on the immune system would benefit different populations—including healthy people, people with metabolic syndromes, and those with other physiological or pathophysiological conditions—is subject to discussion.

In this systematic review, we summarize clinical trials that studied the immunomodulatory effects of IF. All types of subjects were included and divided into different groups including healthy subjects, obese subjects and others, to clarify the cross-effect between IF and subjects under different physiological and pathophysiological situations, including pregnancy, perioperative period，endocrine disease, cancer and autoimmune diseases. The purpose of this systematic review is to analyze and compare current trials on this topic and to provide insight into the possible influence of IF on the immune system.

## 2. Methods

This systematic review was conducted and presented according to the Preferred Reporting Items for Systematic Review and Meta-Analysis Protocols (PRISMA) guidelines (Supplementary Table S1) and Assessment of Multiple Systematic Reviews 2(AMSTAR 2) tools (Supplementary Table S2). Various databases were searched, including Cochrane, PubMed, and Embase, from January 2005 to August 2022. The terms used for the literature research were “time-restricted feeding,” “time-restricted eating,” “intermittent fasting,” “feeding schedule,” “food timing,” “meal frequency,” “compressed feeding,” and “restricted food intake.” These terms were then united with “OR.” In addition, the terms “normal human,” “adult,” “patient,” and “human” were linked with “OR.” The terms “immune,” “immunity,” “immunologic,” “lymphocyte,” “chemokine,” “interleukin,” “C-reactive protein,” “CRP,” “neutrophils,” “oxidative stress,” “oxidative burst,” “inflammatory,” “inflammation,” “immunoglobulin,” “autoimmune,” “lipid peroxidation,” “homocysteine,” “malondialdehyde,” “MDA,” “glutathione,” and “GSH” were united with “OR” and then added together with the aforementioned terms.

The inclusion criteria were as follows: randomized control trials and cohort studies; age > 18 years; one type of IF conducted; and at least one immunomodulatory marker analyzed. Exclusion criteria were as follows: intervention not strictly followed; no fasting procedure included in the intervention; IF combined with other eating interventions, such as liquid diet, protocol; and review articles.

**Figure 2:**
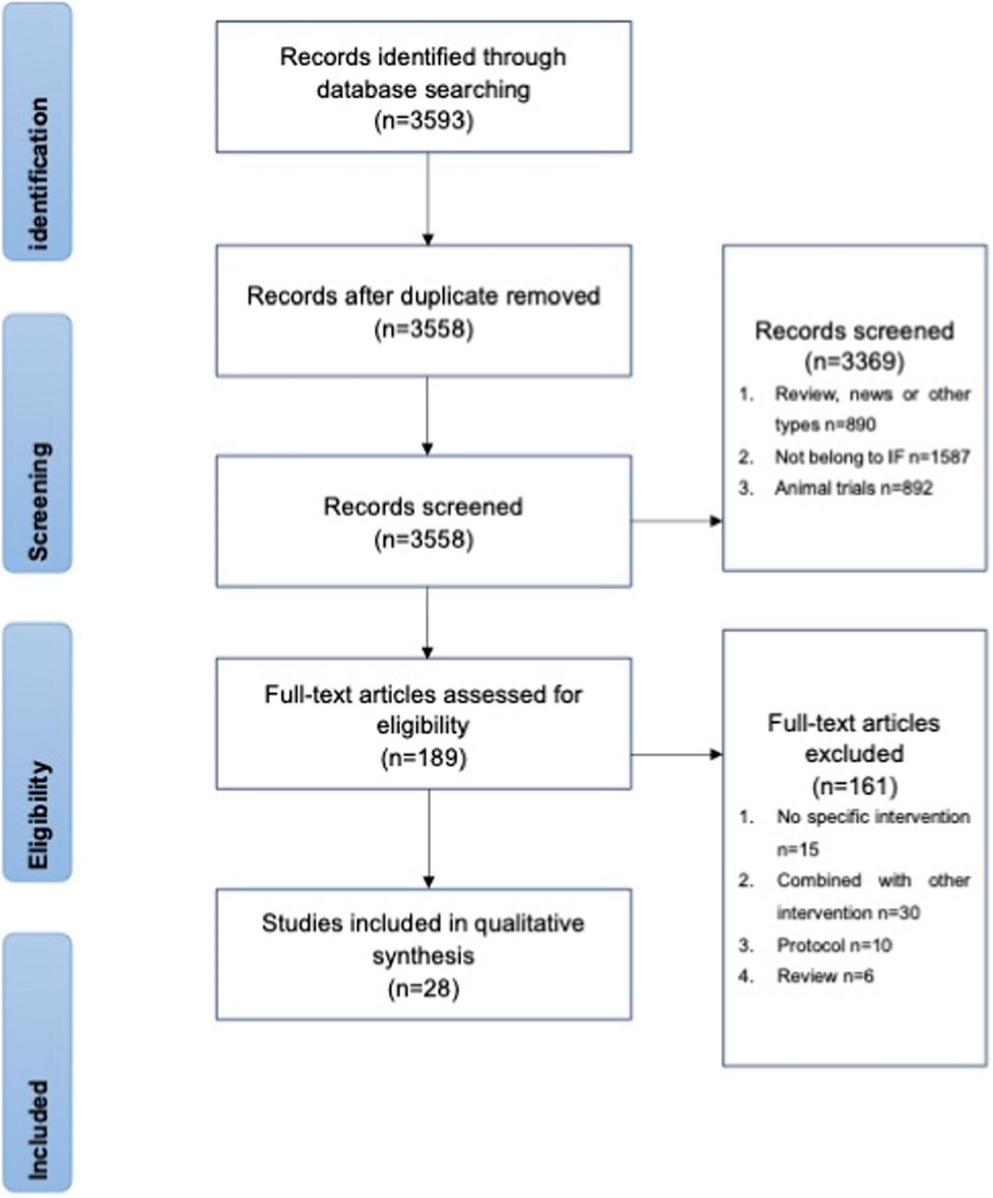
Search and study selection for systematic reviews (PRISMA) flow chart.

A total of 3,558 potentially eligible articles were collected from the databases. After screening, 89 articles were selected for full-text review, of which 61 were excluded for unexpected interventions (Figure 2). Twenty-eight studies were later grouped into effects on healthy people, effects on obese subjects, and effects on other subjects according to the trial set. These grouping procedures were performed by two independent researchers before August 2022. The following parameters were extracted from the original articles for comparison: participants, trial length, intervention, control group, immunomodulatory effect, metabolic information, and body weight.

The Cochrane Collaboration tool (Supplementary Table S3) was applied to assess risk of bias in all included studies. The levels of evidence were as follows: randomized trials, nonrandomized controlled trials, historically controlled cohort studies, and single-arm noncontrolled trials. Because different trials had different levels of bias, a meta-analysis was not performed.

## 3. Results

### 3.1. Effects on non-obese healthy people

Eleven studies measured the immunomodulatory effect of IF on healthy people, and some included assessment of body weight changes or metabolic differences (Table 1).

**Table 1:** Effects on non-obese healthy objects.

Reference	Intervention	Control	Participants	Trial length	Immune immunomodulatory effect	Glucose metabolism	Lipid metabolism	Others	Body weight
Varady et al. (5)	ADF	RCT	BMI26	12w	CRP: ↓(*p* < 0.01) Leptin: ↓(*p* < 0.03) Adiponectin: ↑(*p* < 0.01)		TC: ⌀ LDL: ⌀ HDL: ⌀ TC^*^: ↓(*p* < 0.01)	DBP: ↓ (*p* < 0.05) SBP: ⌀	↓, −6.5 ± 1.0% (*p* < 0.001), on average 5.2 kg.
Wegman et al. (15)	ADF	Crossover	BMI23	3w	Gene-upregulated^*^: *SIRT1, SIRT3, SOD2, TFAM*	Insulin: ↓(*p* = 0.0023)			
Paoli et al. (16)	TRF	Before-after study	resistance-trained male	8w	Adiponectin: ↑(*p* = 0.0000) Leptin: ↓(*p* = 0.0001) IL-1b: ↓(*p* = 0.0235) TT: ↓(*p* = 0.0476) IGF-1: ↓(*p* = 0.0397) IL-6^*^: ↓(*p* = 0.0035) TNF-α^*^: ↓(*p* = 0.0001)	Insulin: ↓(*p* = 0.0303) Glucose: ↓(*p* = 0.0011)	TG: ↓(*p* = 0.0201) HDL: ↑(*p* = 0.0142) LDL: ⌀		↓(*p* = 0.0448)
Lauridsen et al. (3)	IF	Before-after study	lean	4w	TNF-α: ⌀ IL-6: ⌀ IL-10: ⌀ Adiponectin: ⌀ Leptin: ⌀ Cortisol: ⌀	Glucose: ⌀ Insulin: ⌀ HOMAIR: ⌀ HbA1c: ⌀	HDL: ⌀ LDL: ⌀ TG: ⌀ TC: ⌀	ALT: ⌀ SBP: ⌀ DBP: ⌀	↓(*p* = 0.05), on average 1.0 kg.
Gasmi et al. (17)	TRF	RCT	Young and aged	12w	Red cells: ⌀ Monocytes: ⌀ Neutrophils: ↓ White blood cells: ↓ Lymphocytes: ↓ Natural killer cell: ↓				↓ young, (*p* < 0.05)
Madeo et al. (18)	ADF	Cross-sectional	healthy middle-aged	4w	Monocytes: ⌀ Lymphocyte: ⌀ B cell: ⌀ CD4 T cell: ⌀ β-hydroxybutyrate^*^: ↓, (*p* = 0.003)		TC: ↓(*p* = 0.004) HDL: ⌀ LDL: ↓(*p* = 0.011) VLDL: ↓(*p* = 0.009) TG: ↓(*p* = 0.010)	SBP: ↓(*p* = 0.006) DBP: ↓(*p* = 0.0302)	↓, (*p* < 0.0001), on average 3.5 kg.
McAllister et al. (19)	TRF	RCT	BMI28	4w	Cortisol^*^: ↓ Adiponectin^*^: ↑ CRP: ↑	Glucose: ⌀ Insulin^*^: ↑	LDL: ↑ HDL: ⌀ TG: ↓ TC: ↓	SBP: ↑ (*P* = 0.04) DBP: ⌀	
Li et al. (20)	TRF	RCT	healthy man	25d	IL-1β: ⌀ TNF-α: ⌀ Gene-upregulated: *Bmal1*(*p* = 0.0020), *Clock*(*p* = 0.0302), *SIRT1*(*p* = 00068) Microbial richness: ↑ (*p* < 0.005)		TC: ↓ (*p* < 0.0001) TG: ↓(*p* = 0.0052) LDL: ⌀ HDL: ↑(*p* < 0.0001)	AKP: ↓(*p* < 0.009) AST: ↓(*p* = 0.0268) ALT: ↓(*p* = 0.0174) Albumin: ↓, (*p* < 0.0001)	
Moro et al. (9)	TRF	RCT	cyclist	4w	TT: ↓(*p* = 0.0497) CRP: ⌀ ESR: ⌀ IL-6: ⌀ Adiponectin: ⌀ TNF: ⌀ TSH: ⌀ T3: ⌀ Cortisol^*^: ↓(*p* = 0.0005) IGF-1: ⌀	Glucose: ⌀ Insulin: ⌀	TC: ⌀ TG: ⌀	Cr: ⌀	↓,2%(*P* = 0.04)
Paoli et al. (1)	TRF	RCT	healthy	2 m/12 m	TT: ↓(*p* < 0.001) IGF-1: ↓(*p* = 0.039) Adiponectin: ↑(*p* = 0.001) Leptin: ↓(*p* < 0.001) Il-6: ↓(*p* = 0.038) IL-1β: ↓(*p* < 0.001) TNF-α: ↓(*p* = 0.042)	Glucose: ↓(*p* < 0.0001) Insulin: ↓(*p* < 0.0001) HOMA-IR: ↓(*p* < 0.0001)	TC: ⌀(*p* = 0.289) HDL: ↑(*p* < 0.001) LDL: ⌀(*p* = 0.129) TG: ↓(*p* < 0.0001)		↓(*p* = 0.001), on average 2.89 kg.
Mao et al. (21)	TRF	RCT	healthy	5w	TNF-α: ↓(*p* = 0.024) IL-8: ↓(*p* = 0.045) CRP: ⌀ WBC: ⌀ Microbial-diversity: ↑ (*p* = 0.049) Resistin: ⌀ Leptin: ⌀ Ghrelin: ⌀ gene-upregulated: *SIRT1, BMAL1, PER2, SIER1*	HOMA-IR: ↓, (*p* < 0.001, *p* = 0.002) Glucose: ↓(*p* = 0.005)	HDL: ⌀ LDL: ⌀ TC: ⌀ TG: ⌀	SBP: ⌀ DBP: ⌀ AST: ↓(*p* = 0.046) ALT: ⌀ ALP: ⌀ GGT: ⌀	↓(*P* = 0.009), on average 1.6 kg.

WBC, White blood cells; NEUT, Neutrophile Granulocyte; PLT, Platelet; Hgb, hemoglobin; BCR/ABL, BCR/ABL gene; TT, testosterone; CRP, C-reactive protein; IGF-1, Insulin-Like Growth Factor 1; HOMA-IR, Homeostasis model assessment of insulin resistance; IF, intermittent fasting; RCT, randomized control study; CML, chronic myelogenous leukemia; PCOS, polycystic ovary syndrome; TC, total cholesterol; TG, triacylglycerol; LDL, low-density lipoprotein cholesterol; HDL, high-density lipoprotein cholesterol; AST, aspartate aminotransferase; ALT, glutamic-pyruvic transaminase; NK cell, natural killer cell; IL, interleukin; TNF, tumor necrotic factor; BP, blood pressure; ADF, alternative day fasting; TRF, time restricted feeding; TNF-a, tumor necrosis factor-alpha; SBP, systolic blood pressure; DBP, diastolic blood pressure; LDH, lactate dehydrogenase; GGT, gamma-glutamyl transpeptidase; ALP, alkaline phosphatase; HbA1c, glycosylated hemoglobin; VLDL, very low-density lipoprotein cholesterol; Cr, creatinine; ESR, erythrocyte sedimentation rate; TSH, Thyroid Stimulating Hormone; T3, triiodothyronine; ⌀, no significant results; ↑, significantly increasing; ↓, significantly decreasing. Some changes in values and *p*-values are missing as they were not presented in the original manuscript.^*^Indicates that the *p*-value was calculated based on the final and baseline values of participants in the TRF group because no comparison was made between changes in values in a TRF group and a normal diet control group in the original manuscript.

Various parameters were selected to investigate the immunomodulatory effects of IF in the eleven studies. Two studies measured the effects on immune cells but had different results. Madeo et al. found that almost all cell subsets remained the same (18), whereas Gasmi et al. observed that neutrophils, lymphocytes, and natural killer cells changed after a twelve-week trial of IF (17). Several studies have focused on classic inflammatory biomarkers. Lower levels of C-reactive protein (CRP), leptin, and adiponectin were observed in a study by Varady et al. (5). Similar results were reported by Paoli, both in an one-year (long-term) and an 8-week (short-term) trial (1)). However, Lauridsen et al. found that measurements of parameters such as tumor necrosis factor alpha (TNF-α), interleukin 6 (IL-6), and interleukin 10 (IL-10) were not significantly changed after a course of IF (3). Mao et al. reported that lower levels of TNF-α and IL-8 could be observed after 5 weeks of IF (21). A study by Mcallister et al. (19) measured cortisol levels and found no significant change and these results were replicated in a study by Moro et al. (9). Moro et al. also reported a significant decrease in testosterone levels (9). Two studies measured microbial diversity after IF and concluded that IF generated great richness (20). Li et al. attempted to explain this change and found that sirtuin1 (*SIRT1*) expression was higher after IF compared with baseline levels (20), which was regarded as a stimulator for circadian genes and correlated with microbial diversity. A study by Wegman et al. also measured Sirt-1–related genes and reported similar results (15).

In eight trials, the decrease of body weight was observed after several weeks; three additional studies did not assess this factor. With regard to glucose metabolism, seven studies measured levels of fasting insulin and fasting glucose and conducted the test of homeostatic model assessment of insulin resistance (HOMA-IR) (1). Two studies found no significant changes in these parameters (3, 9), whereas improvements in these parameters were observed in five other studies (1, 15, 16, 19, 21).

Nine studies measured parameters related to lipid metabolism, including total cholesterol (TC), triglycerides (TG), low-density lipoprotein (LDL), and high-density lipoprotein (HDL). Six of them found improvements in multiple parameters; IF was associated with higher HDL, lower TC, lower TG, and lower LDL (1, 5, 18, 20). The remaining three studies found no significant changes in these parameters (3, 9, 21). Effects on different parameters, such as systolic blood pressure, diastolic blood pressure, and alanine transaminase, have been reported in other studies (18, 20).

Sleep quality and appetite were evaluated in some studies (5, 19, 21), and there was no significance after IF (21). Another study showed that during fasting, satiety and fullness of subjects were lower than controlled group, but no differences were found in nausea scores between two groups (3). Alertness, focus perceiving and mood perceiving were measured insignificantly in one study (19).

### 3.2. Effects on obese subjects

The effects of IF on obese subjects have received much attention. Twelve studies that assessed this topic were identified (Table 2).

**Table 2:** Effects on obese subjects.

Reference	Intervention	Control	Participants	Trial length	Immune immunomodulatory effect	Glucose metabolism	Lipid metabolism	Others	Body weight
Varady et al. (22)	ADF	Before-after study	Obese	8w	CRP: ⌀ Homocysteine: ⌀ Adiponectin: ↓, −30% (*p* < 0.05) Leptin: ↓, −21 ± 6% (*p* < 0.05) Resistin: ↓, −23 ± 6%		TC 4w: ↓, −20%(*p* < 0.05) LDL 4w: ↓, −31%(*p* < 0.05) HDL 4w: ⌀ TG: 4w ↓, −19%		↓, −3.83%, on average 5.7 kg.
Varady et al. (2)	ADF	RCT	Obese	12 m	CRP: ⌀ Homocysteine: ⌀	Glucose: ⌀ Insulin: ⌀	HDL: ↑	BP: ⌀	^*^↓, −6%
Peterson et al. (23)	TRF	RCT	Prediabetes	5w	8-isoprostane: ↓, −11 pg./ml (*p* = 0.05) TNF-α: ⌀ cortisol: ⌀	Glucose: ⌀ Insulin: ↓(*p* = 0.13)	HDL: ⌀ LDL: ⌀ TC: ↑(*p* = 0.0007)	SBP: ↓, -11 mmHg (*p* = 0.03) DBP: ↓, -10 mmHg (*p* = 0.03)	^*^↓(*p* = 0.12)
Bowen et al. (24)	ADF	RCT	Obese	24w (16w + 82)	CRP: ↓	Insulin: ↓ Glucose: ↓	HDL^*^: ↑ LDL^*^: ↓ TC^*^: ↓ TG^*^: ↓	SBP^*^: ↓ DBP^*^: ↓	^*^↓, on average 11.2 kg.
Haus et al. (5)	ADF	RCT	Obese	24w	Adiponectin: ↓ Leptin: ↓ IL-6: ↑ TNF-α: ⌀	Glucose: ↓, (*p* = 0.031) Insulin: ↓, (*p* = 0.115) HOMA-IR: ↓, (*p* = 0.031)			↓, (*p* < 0.001)
Heilbronn et al. (25)	IF	RCT	Obese	8w	TNF-α: ⌀ IL-6: ⌀ IL-10: ⌀ Macrophage: ↓	HOMA-IR: ↓			↓
Varady et al. (6)	TRF	RCT	Obese	10w	8-isoprostane: ↓(*p* = 0.02) TNF-α: ⌀ IL-6: ⌀	Glucose: ⌀ Insulin: ↓, (*p* = 0.02, *p* = 0.04) Insulin resistance: ↓, (*p* = 0.03, *p* = 0.04)	LDL: ⌀ HDL: ⌀ TG: ⌀	SBP: ⌀ DBP: ⌀	↓,3.2%(4 h) ↓,3.2%(6 h)
Zouhal et al. (26)	IF	RCT	Obese	30d	IL-6^*^: ↓, (*p* = 0.02) TNF-α^*^: ↓, (*p* = 0.019)			AST: ⌀ ALT: ⌀ LDH: ⌀ Urea: ⌀	↓,2.7% (*P* = 0.002)
Mindikoglu et al. (10)	IF	Before-after study	Metabolic syndrome	4w	leptin: ⌀ Adiponectin: ⌀ CRP: ⌀ Homocysteine: ↑ (*p* = 0.0004) IL-1: ⌀ IL-6: ⌀ IL-8: ⌀ TNF-α: ⌀ Gene-upregulated: *AP5Z1, YPS8, INTS6, IGFBP5, POLRMT, KIT, CROCC, PIGR, CALU* Gene-downregulated: *POLK, CD109, SRGN, CAMP*	HOMA-IR: ⌀ Glucose: ⌀ Insulin: ⌀	TG: ⌀ HDL: ⌀ TC: ⌀ LDL: ⌀	SBP: ↓(*P* = 0.023) DBP: ↓(*p* = 0.002) ALT: ⌀ AST: ⌀ GGT: ⌀ ALP: ⌀ Albumin: ⌀	↓(*p* < 0.0001), on average 2,5 kg.
Horne et al. (27)	IF	RCT	Metabolic syndrome	4w/13w/26w	Galectin-3: ↑(*p* = 0.021)				
Heilbronn et al. (28)	IF	RCT	obese women	8w	Gene-nonregulated: *LIPE, ACACA, FASN, DGAT1* Gene-upregulated: *PLIN5* Gene-downregulated: *SOD1, SOD2* β-hydroxybutyrate:↑(*p* < 0.05)				↓(*p* < 0.05)
Safavi et al. (8)	ADF	RCT	Metabolic syndrome	4 m	CRP: ↓(*p* = 0.03) TNF-α: ↓(*p* = 0.60) IL-6: ↓(*p* = 0.49) PT: ↑(*p* < 0.001) APTT: ↑(*p* = 0.04)	Glucose: ↓(*p* = 0.03)			↓(*p* = 0.02), on average 6.43 kg.

WBC, White blood cells; NEUT, Neutrophile Granulocyte; PLT, Platelet; Hgb, hemoglobin; BCR/ABL, BCR/ABL gene; TT, testosterone; CRP, C-reactive protein; IGF-1, Insulin-Like Growth Factor 1; HOMA-IR, Homeostasis model assessment of insulin resistance; IF, intermittent fasting; RCT, randomized control study; CML, chronic myelogenous leukemia; PCOS, polycystic ovary syndrome; TC, total cholesterol; TG, triacylglycerol; LDL, low-density lipoprotein cholesterol; HDL, high-density lipoprotein cholesterol; AST, aspartate aminotransferase；ALT, glutamic-pyruvic transaminase；NK cell, natural killer cell; IL, interleukin; TNF, tumor necrotic factor; BP, blood pressure; ADF, alternative day fasting; TRF, time restricted feeding; TNF-a, tumor necrosis factor-alpha; SBP, systolic blood pressure; DBP, diastolic blood pressure; LDH, lactate dehydrogenase; GGT, gamma-glutamyl transpeptidase; ALP, alkaline phosphatase; HbA1c, glycosylated hemoglobin; VLDL, very low-density lipoprotein cholesterol; Cr, creatinine; ESR, erythrocyte sedimentation rate; TSH, Thyroid Stimulating Hormone; T3, triiodothyronine; PT, prothrombin time; APTT, activated partial thromboplastin time; ⌀, no significant results; ↑, significantly increasing; ↓, significantly decreasing. Some changes in values and *p*-values are missing as they were not presented in the original manuscript.^*^Indicates that the *p*-value was calculated based on the final and baseline values of participants in the TRF group because no comparison was made between changes in values in a TRF group and a normal diet control group in the original manuscript.

Heilbronn et al. found that levels of TNF-α, IL-6, and IL-10 changed insignificantly during 8 weeks of IF, whereas macrophage counts increased significantly (25). Changes in CRP levels have been measured in several trials; however, almost no significant differences were observed (2, 10, 23, 24, 29). Conversely, Varady et al. found that 8-isoprostane decreased after 10 weeks of IF (6); these results were similar to those of a trial by Peterson et al. (23). Haus et al. reported that adiponectin and leptin levels decreased after a course of 24 weeks (29), and these results were confirmed by Varady et al. in a before–after study (22). Significant changes in IL-6 and TNF-α levels were observed in a study by Zouhal et al. (26). After a four-month trial conducted by Safavi et al., subjects had lower CRP levels (8). Mindikoglu et al. attempted to determine the immunomodulatory effects of gene expression like *AP5Z1* after finding almost no significant change on inflammatory parameters (10). Heilbronn et al. also found that gene expression like *PLIN5* may result in immune system changes (28).

Significant body weight reductions were observed in all studies except that of Horne et al., which only identified significant changes in galectin-3 levels (27). Because metabolic syndrome is often related to obesity, glucose and lipid metabolism have been extensively researched in obese subjects. Mindikoglu et al. compared fasting glucose and insulin levels before and after 4 weeks of IF and found no significant changes (10). Varady et al. also found no improvements in glucose metabolism in obese subjects who completed IF, but that study did identify higher level HDL (2). Six studies found that fasting insulin, fasting glucose, and HOMA-IR levels were improved after IF than before (6, 8, 23, 24, 26, 29). Augmentation of lipid metabolism was observed in a study by Varady et al. in obese subjects (22). However, other studies on lipid metabolism did not show such significant results. In addition to the collected metabolic findings, four studies found that IF could reduce blood pressure levels (10, 23, 24, 26).

### 3.3. Effects in other conditions

Five studies focused on the effects of IF on special populations, including individuals in special physiological states, such as during pregnancy or before or after an operation, and individuals with conditions such as polycystic ovary syndrome (PCOS), multiple sclerosis (MS), or chronic myelogenous leukemia (CML) (Table 3).

**Table 3:** Effects in other conditions.

References	Intervention	Control	Participants	Trial length	Immune immunomodulatory effect	Glucose metabolism	Lipid metabolism	Others	Body weight
Ozturk et al. (30)	IF	RCT	Pregnant	4w	Oxidative stress index (OSI): ⌀ Total oxidant status (TOS): ⌀ Total anti-oxidant status (TAS): ⌀				
Nashwan et al. (31)	IF	Retrospective study	CML		WBC, NEUT, PLT, HGB^*^: ⌀ BCR/ABL^*^:⌀				
Bing he et al. (32)	Eating on 8:00–16:00	Before-after study	PCOS	5w	TT: ↓(*p* = 0.048) CRP: ↓(*p* = 0.040) IGF-1: ↑(*p* = 0.006)	Glucose: ⌀ Insulin: ↓ (*p* = 0.017) HOMA-IR: ↓(*p* = 0.025)	TG: ⌀(*p* = 0.715) TC: ⌀(*p* = 0.328) LDL: ⌀(*p* = 0.984)	AST: ↓(*p* = 0.113) ALT: ↓(*p* = 0.027)	↓(*p* < 0.001), on average 1.3 kg.
Fitzgerald et al. (33)	IF	RCT	Obese, multiple sclerosis	8w	Leptin: ⌀ Adiponectin: ⌀ Memory T cell subsets: ↓ Naïve subset: ↑ Th1 cell: ↓				↓
Ginhoven et al. (34)	IF	RCT	Kidney donation，BMI25		CRP: ⌀ WBC, B cell, T cell: ⌀ NK cell: ↓after surgery (*P* < 0.001) IL-10, IL-6: ⌀ TNF-α: ⌀ before surgery, ↓after surgery Cytokine: ⌀ IL-8: ↑(*p* = 0.018)				

WBC, White blood cells; NEUT, Neutrophile Granulocyte; PLT, Platelet; Hgb, hemoglobin; BCR/ABL, BCR/ABL gene; TT, testosterone； CRP, C-reactive protein; IGF-1, Insulin-Like Growth Factor 1; HOMA-IR, Homeostasis model assessment of insulin resistance; IF, intermittent fasting; RCT, randomized control study; CML, chronic myelogenous leukemia; PCOS, polycystic ovary syndrome; TC, total cholesterol; TG, triacylglycerol; LDL, low-density lipoprotein cholesterol; HDL, high-density lipoprotein cholesterol; AST, aspartate aminotransferase；ALT, glutamic-pyruvic transaminase；NK cell, natural killer cell; IL, interleukin; TNF, tumor necrotic factor; BP, blood pressure; ADF, alternative day fasting; TRF, time restricted feeding; TNF-a, tumor necrosis factor-alpha; SBP, systolic blood pressure; DBP, diastolic blood pressure; LDH, lactate dehydrogenase; GGT, gamma-glutamyl transpeptidase; ALP, alkaline phosphatase; HbA1c, glycosylated hemoglobin; VLDL, very low-density lipoprotein cholesterol; Cr, creatinine; ESR, erythrocyte sedimentation rate; TSH, Thyroid Stimulating Hormone; T3, triiodothyronine; ⌀, no significant results; ↑, significantly increasing; ↓, significantly decreasing. Some changes in values and *p*-values are missing as they were not presented in the original manuscript. *Indicates that the *p*-value was calculated based on the final and baseline values of participants in the TRF group because no comparison was made between changes in values in a TRF group and a normal diet control group in the original manuscript.

Ozturk et al. conducted a study of Ramadan IF in pregnant women. Total antioxidant status, total oxidant status, and related indices were measured; however, none showed significant changes after 4 weeks of the intervention. Pregnancy complications and birth weights were measured but showed no significant results between the IF-treated group and the controlled group (30). A study by Ginhoven et al. focused on IF during the perioperative period; 30 subjects who underwent kidney donation surgery were randomly assigned into a 1-day fasting group and a four-day restriction group. Many indicators were examined including CRP, white blood cells (WBCs), B cells, T cells, natural killer cells, IL-10, IL-6, TNF-α, and lipopolysaccharide. No statistically significant preoperative differences between groups were observed, with the exception of IL-8, which peaked at 6 hours after surgery in both groups but was significantly higher in the restriction group (*p* = 0.018). After surgery, the restriction group showed lower natural killer cell counts, lower WBC counts, and lower TNF-α levels (34).

Yassin et al. conducted a retrospective study of the effects of IF in subjects with CML. Forty-nine subjects were enrolled and tested before, during, and after fasting. BCR-ABL expression levels were measured and showed no significant difference among the three time points. Various hematological parameters, including WBC, hemoglobin, and platelet levels, showed no significant changes (31).

An eight-hour IF was conducted in 15 women with PCOS for 5 weeks; participants reported significant decreases in body weight (32). Metabolic parameters were also assessed, and lipid metabolism had insignificant changes, whereas fasting insulin levels and HOMA-IR decreased significantly after IF compared with their baseline levels (32). Total testosterone decreased by approximately 10%, but changes in luteinizing hormone (LH) and follicle-stimulating hormone (FSH) levels were not significant (32). A reduction in high-sensitivity CRP (hsCRP) and alanine transaminase (ALT) levels was observed, and insulin-like growth factor 1 (IGF-1) was upregulated (32). Fitzgerald et al. found that IF could alter T-cell subsets and metabolic markers in subjects with multiple sclerosis. The subjects in that study lost an average of 3.0 kg after the eight-week trial and had no significant changes in leptin and adiponectin levels. Individuals in the IF group showed significant reductions in memory T cells and increased naïve cell subsets (33).

## 4. Discussion

For non-obese healthy people, it is believed that the body could maintain a steady state in which lipid and glucose metabolism are effective and the immune system works well (18). From some perspectives, IF could still benefit healthy people. After IF, WBC subsets in two trials changed in different ways (17, 18). The reduction in neutrophils may have resulted from the migration to extravascular lymphoid tissues (17). This process requires a long intervention, so another shorter trial conducted by Madeo et al. did not show such results (18). More studies showed that the elimination of old damaged cells would process during fasting, and more active immune cells would be generated when fasting ended (35). In this way, IF could protect various tissues against diseases with more active immune cells by hormesis mechanisms that increase cellular stress resistance (36). A decrease in natural killer cells is mainly linked to a decrease in IL-2 or IGF-1. Neither of which were measured in the trial by Madeo et al., but it could be observed in two studies by Paoli et al. (1, 16). Besides IGF-1, other measurements also show significant changes. Adiponectin may interact with adenosine 5′-monophosphate-activated protein kinase (AMPK) (19), which then helps to regulate insulin resistance (9). High level of adiponectin would stimulate fatty acid oxidation in skeletal muscle and inhibit glucose production in the liver, which benefit to energy homeostasis (37). Meanwhile, adiponectin is an anti-inflammatory agent, a reduction of inflammatory markers including CRP and TNF-α could be observed in some studies (1, 5). Changes in gene expression provide more information on immunomodulatory effects: Wegman et al. concluded that an increase in *SIRT1* and sirtuin3 (*SIRT3*) expression could be detected after a 3-week trial (15). For *SIRT1*, other studies have also shown an increase level (21). *SIRT1* is linked to circadian rhythms and cellular mechanisms, such as cell repair, division, metabolism, and growth (20). It could be concluded that IF could protect bodies from cardiovascular diseases. *SIRT3* is a member of the sirtuin family of histone deacetylases, which are primary mitochondrial protein deacetylases. Moreover, it could regulate cell metabolism, thus maintaining myocardial energy steady. *SIRT3* is also believed as a protection for cardiomyocytes from oxidative stress-mediated cell damage (38). Besides that, some animal studies showed more exciting results through *SIRT3* regulation of IF. High expressions of *SIRT3* in cerebral cortical and hippocampal cells could benefit for treating anxiety and cognitive disorders, which was found as considerable overlap mechanisms by which IF and exercise enhance brain function of Alzheimer’s Disease patients (39, 40). A study by Mao et al. investigated clock genes and showed that levels of genes such as *BMAL1* and *PER2* were elevated in a five-week trial (21), indicating that IF could partly modulate the immune system by improving the circadian rhythm. The reinforcement of circadian rhythm could benefit body immune through promoting system recovery and the clearance of harmful cellular element (41). Another potential immunomodulatory effect involves microbial diversity in two studies (20, 21): Low gut microbial diversity is associated with metabolic disease (42), and high diversity may be due to the high expression of *SIRT1* and high levels of HDL (20) and improve body immune system, such as liver function mentioned in the study by Li et al. Emerging evidence showed that SIRT1 could promote gut microbial population shifts by influence inflammation and circadian rhythm (43). It has also been suggested that IF could benefit healthy people lose weight (9), even cyclists and men who practice resistance training (9, 16). After the trial, it was concluded that IF could lose almost fat and maintain muscle mass with the measurement of muscle area of the thigh and arm. Healthy individuals might already have high insulin sensitivity at baseline; thus, IF seems to have less influence on glucose metabolism in these non-obese and healthy individuals (18). Things were similar when focusing on lipid metabolism. A decrease in leptin was found in many studies (1, 16), which might suggest that IF could partly strengthen lipid metabolism in healthy individuals. To sum up, IF could benefit immune system of healthy people through migration of immune cells, regulation of oxidative-related and circadian-related genes, increasing gut microbial diversity and improvement of muscle-fat ratio. Trials with longer durations and more factors including anxiety degree, cognition state, microbial diversity (44–46), key gene expression, and inflammatory markers are needed to better clarify the immunomodulatory effects of IF on healthy people.

Most obese subjects would harbor inflamed adipose tissue, which could cause a persistent, low-grade, inflammatory response. Obesity is often associated with the metabolic syndrome, because fat accumulation would cause insulin resistance (47). And evidence accumulated that persistent inflammation of adipose tissue is a central mechanism through which obesity promotes cancer risk (48). From the perspective of immune cells, A decrease in macrophages was observed in a study by Heilbronn et al. (25). Most cytokines that are produced by adipose tissue originate from nonfat cells and macrophages (29), thus the result confirmed that IF could be beneficial for inflammation associated with obesity. Recent studies have suggested that IF inhibits the nuclear factor kappa-B signaling pathway, which is an important regulator of downstream parameters including TNF-α and IL-6 (25), which is consistent with the results that IF could partly eliminate the inflammation caused by adipose tissue, with lower CRP and TNF-α (26). There were insignificant changes of some inflammatory markers in some studies, which might be related with short trial duration and inadequate weight loss (6). The concentration of galectin-3, which plays various roles in humans, was measured increasingly by Horne et al. in 2021 (27). It has been shown that galectin-3 could stimulate the expression of some antiviral genes and protect against inflammation, which may result in improvements in glucose metabolism. Although changes in inflammatory factors were less significant in obese people compared with healthy subjects, the immunomodulatory effect of IF observed in obese people might reflect a suppression of oxidative stress (26). Heilbronn et al. found that the ketone bodies, especially β-hydroxybutyrate, which protects against lipotoxicity and stimulates lipid oxidation, was significantly elevated in obese subjects (28). As it was regarded as an epigenetic regulator in terms of histone methylation, acetylation, IF could help to delay various age-related diseases. A decrease in 8-isoprostane, a marker of oxidative stress in lipids, was observed in two studies (6, 23). Oxidative stress is a definition of the imbalance between the production and elimination of reactive oxygen species (49). Some other studies have suggested that, though IF might have little effect on inflammation, it may greatly influence oxidative stress, which is linked to insulin resistance (26). Interestingly, improvements in glucose metabolism were observed in two studies that reported decreased oxidative stress markers (6, 23). Significant changes in leptin, which is regarded as a special body weight regulating hormone, were also noted (29), meaning that the resistance to leptin is partly improved in obese subjects. Besides the ability to regulate metabolic syndrome, including lowering glucose and lipid synthesis (50), leptin is one of the mediators responsible for the inflammatory state (51). In addition to the findings about immune cells and inflammatory markers, other study conduct tests of gene expression. Heilbronn et al. found that genes related to oxidative stress were down-regulated such as *SOD1* and *SOD2* (28), and Mindikoglu et al. found that the expression of other genes including the tumor activators *POLK*, *NIFK*, *SRGN*, *CAMP*, and *D109*, were downregulated (10), which are consistent with remitting oxidative effect and lowering cancer risk by IF. To sum up, besides the advantages of IF on obese subjects including losing body weight, regulate lipid metabolism and improve insulin resistance, which was almost suggested in all studies, IF could reduce oxidative stress and remit inflammatory state through macrophage adjustment and hormone secretion. Moreover, although evidence is accumulating that gut microbial is involved in the etiology of obesity (52) and altered by modified IF (4), relevant researches were still rare. Another issue waiting for more studies was the influence between IF and nervous system on obese subjects. Neuroinflammation, which has emerged as a crucial cause of cognitive dysfunction, such as Alzheimer’s Disease, could be caused through inflamed adipose tissue of obesity (53). A study in obese rat showed that IF could prevent memory loss in comparison to *ad libitum* by regulating body metabolism (54), which offering a new sight for the advantages of IF to remit neuroinflammation.

Pregnancy is a state of high oxidative stress, which contributes to preeclampsia and restriction of fetal growth (30). Maternal IF resulted in detrimental influence on fetal development and maternal stress stage by changing the metabolite profiles in animal studies (55). However, IF has no significant influence on the high oxidative stress and fetal development in the human study (30). The reason could be the different circadian rhythms between rats and humans. A case related with gestational diabetes mellitus was reported that IF is a useful intervention to reduce maternal body weight, plasma glucose, and psychological distress without any adverse effects (56). Surgery is regarded as a shock or an acute stress, and IF is able to improve resistance to this stress. In a study by Ginhoyen et al. (34), a higher preoperative IL-8 level may counter the proinflammatory influence of subsequent surgery, thus TNF-α was lower in the food-restriction group after surgery. Compared with subjects in the non-fasting group, subjects in the restriction group showed a more moderate postoperative inflammatory response. For healthy people in special physiological states, such as those observed during the perioperative period, IF could reduce acute stress. More trials are needed to identify the influence on pregnant subjects, including the fetal and maternal safety, anti-stress effect and body metabolism regulation. It is worth nothing that study include in this review on pregnant subjects was a Ramadan IF trial, which might be less convincing as subjects in this study had experienced such interventions before.

PCOS is an endocrine condition closely linked to metabolic disorders. Because obesity is closely related to PCOS, it is not surprising that IF could provide benefits by reducing insulin resistance and easing hyperandrogenemia (32). Whether IF could be applied in subjects with cancer remains unclear (57), because it may also affect chemotherapy. In a study of subjects with CML, Yassin et al. reported that fasting did not result in significant immunological effects with measurements including BCR-ACL levels and hematological parameters (31). It was suggested that IF in some patients who have cancer could be capable of decreasing chemotherapy-related toxicity and tumor growth, however (58), more clinical trials were needed to clarify. MS is an autoimmune disease characterized by degeneration of the central nervous system (59). The epidemiology of this condition includes a history of childhood obesity. Although no significant changes in leptin or adiponectin levels have been observed in studies of IF in MS, an observed difference in T-cell subsets in intestines might explain the immunological effects of IF that have been reported in studies (33, 60), which was also a kind of possible therapy for MS (59). The components of the intestinal microbiome could also raise the propensity to develop MS strongly (59). Researches about gut microbial of the influence of IF on subjects who have MS were expected as a result of migration of intestine immune cell subsets. As mentioned before, IF is beneficial for nervous system by cellular, metabolic and circadian mechanisms and a promising therapy for brain disorders, future research should disentangle whether positive effects of IF could be applied in clinical situations (61). Besides IF, other types of diet, including energy-restricted fasting and ketogenic diet (62), were also evaluated as nutrition therapy for MS (63). Some advantages were concluded that ketone bodies produced in these diets could serve as an alternative energy source for the brain (62), and during 3-day cycles of a fasting mimicking diet, it was found that the clinical symptoms of experimental autoimmune encephalomyelitis mice. More results was put forward that the improvement of this diet was related with immune system, including reducing inflammatory cytokines and immune cell migration. However, these diets might cause deficiency of various nutrients in long term (63). To sum up, a special diet could serve as a unique nutrition therapy for MS with disadvantages of nutrition deficiency, which was nowadays a popular and promising topic.

The different evidence levels should be taken into consideration when analyzing the results of these studies. Of the 28 selected trials, 19 were randomized, controlled, parallel, or crossover studies. Some trials were cohort studies, and the trial focusing on CML was a retrospective study; the lack of a control group in that trial may lead to inaccurate conclusions. Trials differed in terms of baseline characteristics, study durations, meal types, and IF types. These differences may interfere with the final results. For example, Gasmi et al. studied whether young people and old people would act differently while undertaking IF (17), Paoli et al. compared all factors in a 2-month trial and in a 1-year trial (1), and Varady et al. focused on whether the influence of IF would vary with different durations of eating windows (6). In the future, more studies on this topic should be conducted to provide new data.

This systematic review finds substantial evidence that IF can modulate the immune system in non-obese healthy people, obese people, and subjects in other physiological or pathophysiological states and these effects were clinically relevant with cognitive improvement, lipid and metabolism regulation, and inflammatory state remission. The mechanisms influenced and regulated to drive changes in each population differ. For example, non-obese healthy people can metabolize lipids and glucose efficiently, so the immunomodulatory effect is reflected in immune cell subset migration, lower inflammatory factors, upregulation of circadian rhythm–related gene expression, and greater microbial diversity. Although weight reduction has also been observed in healthy people, changes in parameters of lipid and glucose metabolism remained insignificant in most cases. In obese people, IF contributes to body health by regulating macrophages, which is related to the inflammatory stage of adipose tissue. Although many inflammatory factors did not show significant changes in obese subjects, other important factors, including 9-isoprastane, leptin, and galectin-3, had significant changes. The gene expression of cancer activators and lipid oxidative activators provides insights into the mechanisms behind these immunomodulatory effects. In pregnant women, IF seems safe to be conducted and possibly useful to treat endocrine disorders during pregnancy. Moreover, IF is able to improve resistance to the stress of surgery. IF can be beneficial for the immune system of individuals with PCOS by improving endocrine function. Limited trials studying the effects of IF on cancer have been conducted. For nervous system, IF is believed to be applicable to treat anxiety and cognitive disorders by cellular, metabolic and circadian mechanisms. However, more trials are needed to better understand the effects and mechanisms by which IF modulates the immune system.

## 5. Conclusion

Our systematic review, analyzing data from IF studies in different populations, suggests that IF could have immunomodulatory effects in healthy people, obese people, and people with special physiological and pathophysiological conditions. Different mechanisms may contribute to these effects. IF can benefit non-obese healthy individuals by strengthening circadian rhythms, migrating immune cells, lower inflammatory factors, and enriching microbial diversity. In addition of the anti-inflammatory effect by regulating macrophages, protection against oxidative stress with hormone secretion and oxidative-related gene expression plays a key beneficial role for the influence of IF on obese subjects. Physiological stress by surgery and pathophysiological disorders by endocrine diseases may be partly eased with IF. Moreover, IF might be used to treat anxiety and cognitive disorders with its cellular, metabolic and circadian mechanisms. Finally, the specific effects of IF and the mechanisms pertaining to immune system in these conditions require additional studies.

## Data availability statement

The original contributions presented in the study are included in the article/Supplementary material, further inquiries can be directed to the corresponding authors.

## Author contributions

ZH, HX, HY, and YM contributed to conception and design of the study. ZH and HX organized the methodology, investigation, and data collection. ZH and CL performed the statistical analysis. ZH wrote the first draft of the manuscript. All authors contributed to manuscript revision, read, and approved the submitted version.

## Funding

This work was supported by grants from CAMS Innovation Fund for Medical Sciences (CIFMS) (No. 2021-I2M-1-058) and National High Level Hospital Clinical Research Funding (2022-PUMCH-B-034).

## Conflict of interest

The authors declare that the research was conducted in the absence of any commercial or financial relationships that could be construed as a potential conflict of interest.

## Publisher’s note

All claims expressed in this article are solely those of the authors and do not necessarily represent those of their affiliated organizations, or those of the publisher, the editors and the reviewers. Any product that may be evaluated in this article, or claim that may be made by its manufacturer, is not guaranteed or endorsed by the publisher.
