# Supplementary material for: Intermittent fasting and immunomodulatory effects: A systematic review
Source: Front Nutr. 2023 Feb 28;10:1048230. doi: 10.3389/fnut.2023.1048230 (PMC10011094; doi:10.3389/fnut.2023.1048230)
Supplement: Supplementary file 3 [file Table_3.docx]

**Supplementary Table 3** Cochrane tool for quality assessment in included studies

| **Reference** | **Random Sequence generation (selection bias)** | **Allocation concealment (selection bias)** | **Blinding of participants and researchers (performance bias)** | **Incomplete outcome data (attrition bias)** | **Selective reporting (reporting bias)** | **score 5/5 low risk** |
| --- | --- | --- | --- | --- | --- | --- |
| Varady et al (5) | Low | Low | High | Low | Low | 4 |
| Wegman et al(20) | Moderate/High | High | High | High | Low | 1.5 |
| Paoli et al(21) | Moderate/High | Moderate/High | High | Low | Low | 3 |
| Lauridsen et al(3) | Low | Low | High | Low | Low | 4 |
| Gasmi et al(16) | Low | Low | High | Low | Low | 4 |
| Madeo et al(15) | Low | Low | High | Low | Low | 4 |
| McAllister et al(18) | Moderate/Low | Low | High | Low | Low | 3.5 |
| Li et al(19) | Moderate/Low | Moderate/Low | High | Low | Low | 3 |
| Moro et al(9) | Moderate/Low | Low | High | Low | Low | 3.5 |
| Paoli et al(1) | Moderate/Low | Moderate/Low | High | low | low | 3 |
| Mao et al(17) | Low | Low | High | Low | Low | 4 |
| Varady et al(26) | Moderate/Low | Moderate | High | Low | Low | 2.5 |
| Varady et al(2) | Moderate/Low | Moderate | High | Low | Low | 2.5 |
| Peterson et al(25) | Moderate/Low | Low | High | Low | Low | 3.5 |
| Bowen et al(24) | Moderate/Low | Low | High | Low | Low | 3.5 |
| Haus et al(5) | Low | Low | High | Low | Low | 4 |
| Heilbronn et al(22) | Moderate/Low | Moderate/Low | High | Low | Low | 3 |
| Varady et al (6) | Low | Low | High | Low | Low | 4 |
| Zouhal et al (27) | Moderate/High | High | High | High | Low | 1.5 |
| Mindikoglu et al (10) | Moderate/High | Moderate/High | High | Low | Low | 3 |
| Horne et al (29) | Low | Low | High | Low | Low | 4 |
| Heilbronn et al(28) | Low | Low | High | Low | Low | 4 |
| Safavi et al(8) | Low | Low | High | Low | Low | 4 |
| Ozturk et al (30) | Moderate/Low | Moderate | High | Low | Low | 2.5 |
| Nashwan et al (32) | Moderate/Low | Moderate | High | Low | Low | 2.5 |
| Bing he et al (33) | Moderate/Low | Low | High | Low | Low | 3.5 |
| Fitzgerald et al (34) | Moderate/Low | Moderate | High | Low | Low | 2.5 |
| Ginhoven et al (31) | Moderate/Low | Moderate | High | Low | Low | 2.5 |
